# Progesterone improves the number and quality of hormone induced Fowler toad (*Bufo fowleri*) oocytes

**DOI:** 10.1186/1477-7827-4-3

**Published:** 2006-02-01

**Authors:** Robert K Browne, Hong Li, Jessica Seratt, Andrew Kouba

**Affiliations:** 1 Memphis Zoo, 2000 Prentiss Place, Memphis, TN 38112, USA; 2 Department of Biology, University of Memphis, Memphis, TN 38152, USA; 3 Shanghai Zoo, Shanghai, 200335, Peoples Republic of China

## Abstract

Combinations of progesterone, lutenizing hormone releasing hormone analogue (LHRHa), human chorionic gonadotrophin (hCG), and the dopamine-2 (DA2) receptor antagonist 1-[1-[4,4-bis(4-Fluorophenyl)butyl]-4-piperidinyl]-1,3-dihydro-2H-benzimidazol-2-one (Pimozide; Orap) were tested for improvement of spawning rates, oocyte numbers, fertilization and neurulation rates of the Fowler toad (Bufo fowleri). Only treatments combined with progesterone produced large numbers of oocytes. The best treatment on oocyte numbers, neurulation rates, and the number of neurulas was with 5 mg progesterone, 20 mic.g LHRHa, and 0.25 mg Pimozide. Progesterone (5 mg) with 60 mic.g LHRHa gave high spawning rates, oocyte numbers, and fertilization rates but neurulation rates were low. Progesterone alone in high repeated doses did not result in ovulation. High doses of LHRHa (60 mic.g) with hCG, progesterone, and Pimozide gave the greatest number of toads spawning, however, they resulted in low oocyte numbers, fertilization and neurulation rates. A low dose of LHRHa (4 mic.g) with hCG, or hCG alone as a second administration, and progesterone with Pimozide produced few good quality oocytes. Toads were given normal ovulatory doses of hormones 24 or 48 hrs after their initial dose, but these resulted in low oocyte numbers followed by poor fertilization. Overall, these results suggest that progesterone with a dose between 20 mic.g and 60 mic.g of LHRHa may be optimal for the induction of ovulation in these toads. Moreover, Pimozide can supplement low doses of LHRHa but not replace it.

## Background

Captive breeding and genetic resource banking [1,2] are necessary to maintain the viability of the approximately 32% of the worlds amphibian species that are endangered or threatened [3]. The efficient use of captive breeding and genetic resource banks requires the reliable production of oocytes, usually through hormonal induction [1]. The use of either pituitaries or hormone analogues to induce ovulation of amphibians is well established in a few laboratory species of anurans including the African clawed toads (*Xenopus *spp.), northern leopard frogs (*Rana pipiens*), and the wood frog (*Rana sylvatica*) [4,5]. Unfortunately, these established protocols for hormonal induction are often species-specific [5,6], vary with the seasonal maturation of females [7,8], or with husbandry conditions [9] and are limited in their application. Considering that amphibians have a high diversity of reproductive strategies it would be expected that their physiological responses to induction protocols would be correspondingly diverse [10]. Consequently, there is a need to increase the reliability of protocols using hormones for the induction of ovulation in amphibians and to extend the range of these protocols to novel species [1,10].

The two common hormones used to induce ovulation in anurans are human chorionic gonadotrophin (hCG) and luteinizing hormone releasing hormone analogue (LHRHa) [10]. In *Xenopus *spp. hCG is reliable in inducing ovulation, however, it is successful only in a minority of other species [11]. LHRHa as an ovulatory agent has proven more universal among amphibians by stimulating leutinizing hormone release from the pituitary which then results in ovulation and spawning [12]. Of the various forms of LHRHa the synthetic hormone D-Ala6, des-Gly10, ethylamide LHRHa has proved superior to mammal, avian, or fish LHRHa [10].

The ability of single doses of hormones to ovulate anurans is dependent on the presence of mature follicles. These mature follicles are only found in anurans after specific environmental conditions [10]. We have recently completed studies that showed in the Wyoming toad (*Bufo baxteri*) that the sequential administration of a combination of hCG and LHRHa a week prior to ovulation resulted in increased numbers of spawning toads, oocyte numbers, fertilization and neurulation rates [unpublished data]. In addition, both *in-vitro *[13,14] and *in-vivo *[15] studies of the long-term administration of progesterone have increased the number of mature oocytes and their fertility [16,17]. In nature progesterone is released by the follicles to mature stage IV oocytes. This response is also promoted by the artifical application of hCG, or gonadotrphin releasing hormone GnRH produced in response to elevated levels of LHRH [10].

There have been a number of other compounds, in combination with reproductive hormones, used to improve oocyte production and quality in fish, including anti-dopaminergic compounds and saccharides [18,19]. The central dopaminergic system is an important inhibitory component in the regulation of LHRH by the brain [20]. Studies in fish have shown that stress-induced corticosteroids can inhibit the release of gonadotropin to the extent that administration of the dopamine-2 (DA2) receptor antagonist Pimozide™ is needed to achieve high rates of ovulation and oocyte quality [18,20-22]. In Catfish (*Heteropneustes fossilis*), the use of Pimozide™ in the early spawning phase potentiated the otherwise anovulatory dose of 0.05 g GnRH analogue to induce aseasonal ovulation [20]. The mechanism of this response was further studied in the goldfish where Pimozide™ caused increases in serum gonadotropin levels and accumulation of GnRH in the pituitary. This response was inhibited by the centrally active dopaminergic agonist apomorphine [22]. The use of Pimozide™ could also have similar beneficial effects on ovulation rates in captive anurans.

The purpose of this study was to test the ovulatory response, and subsequent spawning, fertilization and neurulation rates of *B*. *fowleri *stimulated with combinations of progesterone, hCG, LHRHa, and Pimozide™.

## Methods

### Animals

Male and female *B. fowleri *were collected in Memphis, Tennessee, USA, (35°15'N,90°05'W) in late July 2005. *B. fowleri *naturally spawn in Memphis from May through July. Toads were housed in 50 × 40 × 10 cm plastic boxes with a substrate of 25 mm thick sponge matting and a piece of cork bark for shelter with a water bowl at one end. The tanks were washed three times weekly, and the toads fed enriched and Reptivite™ (Zoo Med, San Luis Obispo, CA, USA) vitamin supplemented mealworms [23]. The toads were randomly assigned to different treatments and there was no significant (P < 0.05) difference in weight or length of female toads between treatments (pooled weight n = 47, 32.8 ± 1.7, mean ± SD).

### Hormonal induction of spermiation

To induce spermiation, ten male toads were administered a dose of 300 IU of hCG (Sigma-Aldrich, St. Louis, C-1063) dissolved in 100 μl of reagent grade water by injection in the intra-peritoneal cavity. The toads were then placed individually in 3.8 L plastic boxes with 1 cm of tap water. The water was changed if soiling was apparent. To collect spermic urine, toads were carefully removed from their box and superficial water dried with a tissue. The toads were then held by a thumb and index finger, anterior and across the pelvic girdle, above a 150 mm diameter Petri dish. Gentle massaging then promoted urination, usually within 60 sec. Spermic urine was stored in an ice slurry at 0°C until use [24].

### Hormonal induction of spawning and the in-vitro fertilization of eggs

Female toads were randomly divided into seven treatment groups with seven animals per treatment except one group of five. Each treatment group was administered a different combination of hormones and Pimozide™ as outlined in table one. Twenty four or forty-eight hours after the first dose a second dose of hormones were administered to see if further oocyte production could be achieved (Table 1). The hormones tested in various combinations included hCG (Sigma-Aldrich, St. Louis, C-1063), D-Ala6, des-Gly10, ethylamide LHRHa (Sigma-Aldrich, St. Louis, L4513), progesterone (Sigma-Aldrich, St. Louis, P8783), and the anti-dopaminergic compound 1-[1-[4,4-bis(4-Fluorophenyl)butyl]-4-piperidinyl]-1,3-dihydro-2H-benzimidazol-2-one (Pimozide™; Orap^®^; Sigma-Aldrich, St. Louis, P1793). All the combinations of hormones or Pimozide™ were administered through intra-peritoneal injection in 100 μl of phosphate buffered saline (PBS) (Intervet, Millsboro, De 19966, USA) except for those containing progesterone which were administered in 1 ml of PBS. The doses of compounds per. gram of frog weight are listed in Table 2.

**Table 1:** Toads were randomly placed into seven treatments where hormones were administered alone or in variouscombinations. Treatment 7* was administered two doses of progesterone 12 hrs apart. Then all seven treatments were administered a second dose of compounds either 24 or 48 hrs later and evaluated as a second set of treatments (8–14).

Treatment	No. of animals	Hormones administered (First combination)	Period (hrs)	Treatment	Hormones administered (Second combination)
1	7	500 IU hCG + 4 μg LHRHa	24	8	500 IU hCG + 4 μg LHRHa
2	7	20 μg LHRHa	24	9	60 μg LHRHa
3	7	20 μg LHRHa + 0.25 mg Pimozide™+ 5 mg progesterone	24	10	60 μg LHRHa
4	7	20 μg LHRHa + 0.25 mg Pimozide™	24	11	60 μg LHRHa
5	7	60 μg LHRHa + 5 mg progesterone	24	12	60 μg LHRHa
6	7	60 μg LHRHa + 5 mg progesterone + 0.25 mg Pimozide™ + 500 IU hCG	24	13	500 IU hCG
7*	5	5 mg progesterone > 12 h r > 5 mg progesterone	48	14	60 μg LHRHa

**Table 2:** The different doses of hormones or Pimozide™ used in this study and the amountper. gram of toad weight.

Compound	Administered dose	Amount per gram of toad weight
hCG	500 IU hCG	15 IU
LHRHa	4 μg	0.12 μg
LHRHa	20 μg	0.61 μg
LHRHa	60 μg	1.8 μg
Progesterone	5 mg	0.15 mg
Pimozide™	0.25 mg	0.0076 mg

Animals from all treatment groups were placed individually into single 11.4 L plastic boxes containing 1.5 cm of Simplified Amphibian Ringer solution (SAR; 6.6 gl^-1 ^NaCl, 0.147 gl^-1 ^CaCl_2_, 0.149 gl^-1 ^KCl, 0.302 gl^-1 ^NaHCO_3_; 220 mOsmol kg^-1^; [25]) to lengthen the time the eggs would remain fertilizable. The SAR was changed if soiling was apparent. The presence of eggs was then monitored for 24 hrs after injection. After spawning, individual strings of oocytes containing 20 to 30 oocytes were separated into replicates totaling 150–250 oocytes and placed in separate Petri dishes. Approximately 100 μl of urine containing sperm samples of good motility and concentration were then pipetted and gently mixed into the oocytes. The oocytes and sperm were allowed to co-incubate for 10 minutes before flooding the dishes with water. Fertilization rates were tested 6 to 8 hrs post fertilization at larval stage 3–6 Daudin, 1956 [26]. Counts of fertilized eggs and unfertilized oocytes, and neurulas were taken under stereo dissecting microscope (Omano™) and fertilization or neurulation (stage 26–28 Daudin, 1956) rates (%) calculated.

## Results and discussion

This study investigated spawning rates, oocyte numbers, and fertilization and neurulation rates of *B*. *fowleri *in response to stimulation with various combinations of the three hormones, progesterone, LHRHa, hCG and with the hormone agonist Pimozide™ (Table 3,4). Repeated doses of progesterone alone or 20 μg LHRHa alone did not promote a spawning response in any toads. The normal ovulatory dose of 500 IU hCG even when supplemented with 4 μg LHRHa only promoted spawning in two toads and 20 μg of LHRHa plus Pimozide™ only promoted spawning in one toad. Spawning rates above 50% were only found in treatments containing progesterone. For hormone treatments containing progesterone, spawning occurred in 58–85% of the toads. However, progesterone alone in repeated doses did not result in ovulation, showing that under these circumstances progesterone is not an ovulatory hormone. The best response of oocyte numbers per. spawning toad was with 5 mg progesterone, 20 μg LHRHa, and 0.25 mg Pimozide™ (2486 ± 825). This number of oocytes was not significantly different (P < 0.5) from 5 mg progesterone with 60 μg LHRHa (2004 ± 196) or progesterone, 60 μg LHRHa, Pimozide™, and 500 IU hCG (1078 ± 502)(means ± SE). Therefore, treatments with progesterone and an ovulatry hormone gave the highest number of oocytes per. spawning toad. Consequently, progesterone appeared to synergize with LHRHa to promote spawning and to increase oocyte numbers.

**Table 3:** Variables measured in response to different hormone treatments including toad length, weight, the number and percentage of spawning toads, the number of oocytes, percent fertilization, number of neurulas, percent neurulation, and total number of neurulas. Treatment 7* was administered two doses of progesterone 12 hrs apart. Data in the table are expressedas Means ± SE and ranges of spawning toads.

Treatment	Treatment	Length (mm)	Weight (g)	No. and percent of toads spawning	No. oocytes per. spawning toad	Total oocytes	Percent fertilization	Neurulas	Percent neurulation	Total neurulas
1	500 IU hCG + 4 μg LHRHa	63.5 ± 6.7	48.0 ± 18.0	(2/7) 29%	2283 ± 269	4566	0%	0	0%	0
2	20 μg LHRHa	69.4 ± 8.5	50.2 ± 17.9	(0/7) 0%	NA	0	NA	NA	NA	NA
3	20 μg LHRHa + 0.25 mg Pimozide™ + 5 mg progesterone	70.0 ± 8.0	48.8 ± 11.4	(4/7) 58%	2486 ± 825 (350–4140)	9944	35 ± 11% (0–69%)	546 ± 60 (0–1131)	22 ± 12% (0–53%)	2184
4	20 μg LHRHa + 0.25 mg Pimozide™	65.9 ± 4.5	43.5 ± 5.7	(1/7) 14%	627	627	34%	157	25%	157
5	60 μg LHRHa + 5 mg progesterone	67.1 ± 4.7	46.0 ± 6.1	(5/7) 71%	2004 ± 196 (1489–2625)	10020	73 ± 5% (57–89%)	378 ± 106 (0–1081)	19 ± 11% (0–60%)	1890
6	60 μg LHRHa + 5 mg progesterone + 0.25 mg Pimozide™ + 500 IU hCG	65.1 ± 5.0	43.2 ± 7.2	(6/7) 85%	1078 ± 502 (80–3470)	6468	20 ± 8% (0–47%)	129 ± 31 (0–178)	12 ± 5% (0–34%)	774
7*	5 mg progesterone then after 12 hrs another 5 mg progesterone	64.0 ± 2.8	40.0 ± 5.3	(0/5) 0%	NA	0	NA	NA	NA	NA

**Table 4:** Variables measured in response to a second administration of hormones 24 to 48 hrs after the first set of treatments include the number and percentage of spawning toads, the number of eggs, percentage fertilization, number of neurulas, percentage neurulation, and total number of neurulas. Data in the table are expressed as Means ± SE and ranges of spawning toads.

Treatment	Treatment	% Spawn	No. oocytes	% cleavage	Neurulas	% neurulation	Total neurulas
8	500 IU hCG + 4 μg LHRHa	0/7 0%	NA	NA	NA	NA	NA
9	60 μg LHRHa	0/7 0%	NA	NA	NA	NA	NA
10	60 μg LHRHa	0/7 0%	NA	NA	NA	NA	NA
11	60 μg LHRHa	1/7 14%	200	87%	0	0%	0
12	500 IU hCG	3/7 42%	68 ± 50 (13–110)	0%	0	0%	0
13	500 IU hCG	2/7 29%	160 ± 28 (60–100)	0%	0	0%	0
14*	60 μg LHRHa	0/5 0%	NA	NA	NA	NA	NA

Only treatments with LHRHa (20 μg or more) plus another hormone resulted in fertilized eggs. The treatment of 5 mg progesterone with 60 μg LHRHa gave a significantly (P < 0.05) higher percentage of fertilized eggs (73 ± 5%) than either of the other treatments with progesterone; progesterone with 20 μg LHRHa, and Pimozide™ (35 ± 11%); or progesterone with 60 μg LHRHa, Pimozide™, and 500 IU hCG (20 ± 8%)(means ± SE). LHRHa without progesterone resulted in the spawning of only one toad with a fertilization rate of 34%. All the treatments with Pimozide™ produced oocytes. However, progesterone with 20 μg LHRHa yielded a greater percentage of fertilized eggs than the same treatment with Pimozide™. Hence, the ability of Pimozide™ to improve the hormonal induction of anurans is uncertain.

Neurulation rates did not correspond with fertilization rates. The treatment with progesterone and 60 μg LHRHa had the highest fertilization rate (73 ± 5%) but the neurulation rate was similar (19 ± 11%) to that from progesterone, 20 μg LHRHa, and Pimozide™ (22 ± 12%). The highest total number of neurulas (2184) was produced from progesterone, 20 μg LHRHa, and Pimozide™. These results indicate that the effect of Pimozide™ on the different stages of spawn rate, oocyte numbers, fertilization rate, and neurulation rate are inconsistent. Nevertheless, the use of Pimozide™ with progesterone did result in the highest number of neurulas.

Toads were given a normal ovulatory dose of hormones 24 or 48 hrs after their initial dose, but these resulted in zero or few oocytes produced of low quality (Table 4). These results correspond with others where a second dose from 24 to 48 hrs is seldom successful [[10], Browne et al., unpublished data].

From the results of previous studies, the inability of hCG and LHRHa without progesterone, to induce substantial numbers of oocytes or high fertilization and neurulation rates suggests that the *B*. *fowleri *in this study did not initially possess many oocytes at a suitable stage for immediate maturation [27,28]. The females in this study were all above the minimum spawning size for *B*. *fowleri *and were captured at the end of the breeding season in Memphis. Thus, it is probable that many if not all of these toads that were capable of spawning had done so. Studies have shown that previtellogenic oocytes (stage I, II) exist in anurans throughout the year and quickly mature to stage IV oocytes prior to ovulation [27,28]. Immediately after spawning, the stage IV oocytes decline in numbers and require several months of recruitment and maturation prior to establishing a stable population of preovulatory follicles. The apparent ability, shown in this study, of progesterone to mature stage I, II oocytes to stage IV oocytes should also increase oocyte numbers and their viability in anurans kept under conditions which have not resulted in the entrainment of follicle maturity. The use of progesterone should also promote greater oocyte numbers in anurans with mature follicles, as even with natural spawning there are a substantial number of stage I and II immature oocytes [27,28]. However, the dose of progesterone needed to mature oocytes could depend on the degree of follicle maturation. With the ovulation of northern leopard frogs reduced doses of progesterone in combination with pituitaries was required for the induction of ovulation as the breeding season progressed [29] Further assessment of the relationship between dosage of progesterone and the degree of follicular maturation in improving oocytes numbers and quality with a seasonal reproducing species could advance captive breeding protocols for endangered amphibians. In anurans, some studies have shown success in the non-invasive assessment of gonadal maturation through ultrasound [30], although others have considered the invasive method of biopsy more accurate [31]. In fish biopsy performed through the cloaca is the standard method for assessing gonadal maturity [32].

The substantial number of oocytes not cleaving, and the subsequent failure of many cleaved eggs to develop to neurulation could be the result of a high percentage of degenerate oocytes in the post-spawning females. Concomitant with the loss of stage VI oocytes during the spawning season there is an increase in the amount of degenerated (atresic) oocytes from the normal levels found throughout the rest of the spawning cycle [27,28] due to declines in gonadotrophin levels [33-35]. This pattern of the seasonal availability of oocytes could explain the need for progesterone as a maturation promoter to achieve oocyte production and improve oocyte quality. Moreover, this might explain the inability of the ovulating hormones LHRHa and hCG alone to achieve ovulation [27]. Previous studies have shown that exogenous progesterone can result in egg maturation both *in-vivo *and *in-vitro *[13,15,16]. Yet, in this study progesterone alone did not result in spawning. It has also been shown in some other studies, that progesterone alone or in high repeated doses did not result in ovulation and may inhibit subsequent ovulation [13,16].

The highest numbers of oocytes were produced by a combination of progesterone with 20 μg LHRHa and Pimizide™. A combination of 20 μg LHRHa alone or LHRHa with Pimozide™ failed to induce substantial oocyte production and the highest number of oocytes were produced from the three progesterone with LHRHa treatments. This suggests that progesterone matured oocytes within 15 hrs of administration, thus, enabling LHRHa to initiate ovulation. An interesting result of this study was the poor response of all treatments to the normal ovulatory dose of hCG or LHRHa given after 24 or 48 hrs. *In-vitro *studies have also shown that the oocytes of unstimulated *Xenopus laevis *females when exposed to progesterone require approximately 3 to 15 hrs to complete meiotic maturation [13]. These results match those of fish hatcheries where after the initial ovulatory dose of hormones a second dose at 24 hrs is seldom successful (Browne unpublished). The decreased oocyte numbers and fertilization rate when hCG (500IU) was combined with a high dose of LHRHa (60 μg) in comparison to 60 μg of LHRHa alone, agrees with another study where high doses of hormones resulted in poor oocyte quality [10]. An interesting result was the production of oocyte in one animal with 20 μg LHRHa and 0.25 mg Pimozide™ when the same dose of LHRHa alone did not result in oocyte production. Unfortunately, because of the limited number of animals Pimozide™ was not tested with 60 μg of LHRHa. Further studies with high doses of LHRHa in combination with progesterone and Pimozide™ but without hCG could yield superior oocyte production and quality to those tested in this study.

## Conclusion

The results of this study have demonstrated the importance of progesterone administration for final oocytes maturation prior to or simultaneous with hormonal induction of ovulation with LHRHa in potentially aseasonal animals. This combination of hormones promises to increase the efficiency of aseasonal artificial ovulation of captive anurans without adequate environmental entrainment. Furthermore, *in-vitro *fertilization studies could also benefit from the provision of more oocytes of greater viability, especially in cases where the genetic management of small captive populations of endangered amphibians is crucial.

A major limiting factor in the use of hormones and other compounds for the artificial ovulation of anurans in captivity is a scarcity of stage II to IV oocytes [6,27]. The development of these oocytes is entrained seasonally in many species or in other species by stoichasitic rainfall events [27]. Therefore the limited reproductive success for many anuran species in captivity, even with dedicated husbandry programs, demands the creation of hormonal protocols for artificial ovulation which circumvent the natural processes of oocyte recruitment [10]. The results of this study have demonstrated the importance of progesterone administration for final oocyte maturation *in-vivo *prior to, or simultaneous with, hormonal inducation of ovulation with LHRHa in potentially aseasonal animals. This combination of hormones promises to increase the efficacy of aseasonal artificial ovulation of captive anurans without adequate environmental entrainment. Furthermore, *in-vitro *fertilization studies could also benefit from the provision of more oocytes of greater viability, especially in cases where the genetic management of small captive populations of endangered amphibians is crucial. The ability to promote final maturation of oocytes using hormone therapy is particularly valuable considering hibernation protocols for captive amphibians can often cause bacterial and fungal infections that lead to death.

Our results suggest that progestone can mature oocytes from previously spawned females and in combination with LHRHa (20–60 μg) may be optimal for the induction of ovulation, spawning, and the production of fertilizable oocytes. Moreover, these results also suggest that if sequential doses of hormones using progesterone are to mature oocytes they may have to be implemented more than 24 hrs and perhaps more than 48 hrs before the final ovulatory dose. Further studies in this area are warranted and are being investigated by our laboratory.

A promising technology for the aseasonal maturation of oocytes is the use of implants to release hormones in a controlled manner [36,37]. Two methods commonly used for the sustained release of hormones are cholesterol implants for both steroids and proteins [36] and mini-pellets for proteins including LHRHa [37]. The sustained administration of progesterone *in-vivo *has already been shown to increase the maturation of oocytes and its potential to increase the number of mature oocytes in amphibians through slow release implants should be investigated. The use of other hormone agonists besides Pimozide™ or the identification of unknown agents in pituitaries could also hold promise [18,20].

## Authors' contributions

RB designed and coordinated this study. RB, JS, and HL carried out the husbandry of the toads, hormonal induction, and the subsequent collection of data. RB, AK, HL wrote and edited the manuscript. All authors read and approved the final manuscript.
